# The SHOCT Domain: A Widespread Domain Under-Represented in Model Organisms

**DOI:** 10.1371/journal.pone.0057848

**Published:** 2013-02-25

**Authors:** Ruth Y. Eberhardt, S. Josefin Bartholdson, Marco Punta, Alex Bateman

**Affiliations:** 1 European Bioinformatics Institute, Wellcome Trust Genome Campus, Hinxton, United Kingdom; 2 Wellcome Trust Sanger Institute, Wellcome Trust Genome Campus, Hinxton, United Kingdom; Universita' di Padova, Italy

## Abstract

We have identified a new protein domain, which we have named the SHOCT domain (**Sh**ort **C**-**t**erminal domain). This domain is widespread in bacteria with over a thousand examples. But we found it is missing from the most commonly studied model organisms, despite being present in closely related species. It's predominantly C-terminal location, co-occurrence with numerous other domains and short size is reminiscent of the Gram-positive anchor motif, however it is present in a much wider range of species. We suggest several hypotheses about the function of SHOCT, including oligomerisation and nucleic acid binding. Our initial experiments do not support its role as an oligomerisation domain.

## Introduction

Protein domains are regarded as the structural and functional components of proteins. These domains are globular, independently folding units. Many proteins also contain motifs, which are shorter structural elements [1]. Proteins with shared domains may be both evolutionarily and functionally related.

One of our goals in the Pfam database of protein domains and families is to increase our coverage of known protein sequences [2]. One strategy, which we have been using in order to achieve this, is to identify all the sequences that do not already contain a Pfam domain from selected bacterial proteomes and perform iterative searches using these sequences. In some cases the results of these searches enable us to build a new family, and in others they enable us to improve an existing family.

In this paper we describe the identification and preliminary analysis of a domain that is prevalent in bacteria. This domain was discovered in a protein from a Firmicute, *Anaerotruncus colihominis* DSM 17241, a bacterium that was isolated from Human faeces and may be of clinical significance [3], [4]. This bacterium was selected for study because the complete proteome is available and, at the time, the Pfam coverage of this bacterium was relatively low compared to that of related bacteria such as species of *Clostridium* and *Ruminococcus*.

## Methods

### Identification

We initially identified the SHOCT domain using the Jackhmmer program, which forms a part of the HMMER3 package written by Sean Eddy (http://hmmer.janelia.org/) [5]. Jackhmmer is an iterative Profile hidden Markov model search that is capable of detecting remote protein homologues. An iterative search was performed on the uncharacterised protein ANACOL_03317 from *Anaerotruncus colihominis* DSM 17241 (UniProtKB accession B0PET9.1) using the default parameters against the UniProtKB database [6]. To create the entry deposited in Pfam we took a set of representative sequences from the Jackhmmer search and created a seed alignment of 751 sequences. A hidden Markov model (HMM) was then constructed from this seed alignment using the HMMER package hmmbuild program. This HMM was searched against the complete UniProtKB sequence database as described above, using an inclusion sequence threshold of 24.0 bits and, in order to increase sensitivity in detection of multiple copies of the SHOCT domain, the domain inclusion threshold was set at 15.4. Sequence and domain thresholds are discussed in detail in Punta *et al*. 2012[7].

### Cloning and Protein Expression

DNA sequences encoding a SHOCT domain F0QBY7.1 and a randomly shuffled control were created by primer annealing (Tables S1 and S2). Fifteen Àl each of 100 ÀM forward and reverse primers were mixed with an annealing buffer (100 mM Tris, pH 7.5, 500 mM NaCl, 10 mM EDTA plus 10% (v/v) DMSO) and heated to 95°C for 5 minutes then cooled by 0.1°C per minute until 25°C. This yielded annealed products of 93 bp flanked by Not1 and Asc1 restriction sites, which were ligated using T4 ligase (Roche) into a modified pTT3 vector [8] containing a mouse signal peptide [9] followed by 6His-linker-Bio-tag and rat Cd4d3+4 N-terminally of the inserts. The Cd4d3+4-peptide constructs were expressed by transient transfection (as described in [8]) in HEK293E cells grown in Freestyle media (Invitrogen) supplemented with a 1% penicillin-streptomycin solution (10 000 units penicillin, 10 mg/ml streptomycin; Sigma), 50 Àg/ml gentamicin sulphate (Sigma), and 1% FBS (Sigma). For enzymatic biotinylation the constructs were co-transfected with a modified biotin ligase, BirA [10].

### SDS-PAGE and Western blotting

To analyse protein expression, cell supernatants were resolved by SDS-PAGE under reducing conditions using NuPAGE 4–12% Bis Tris precast gels (Invitrogen). Proteins were blotted onto PVDF membranes (Amersham) and blocked in 2% BSA. Membranes were incubated with peroxidase-conjugated streptavidin (Jackson Immuno Research), washed in 1×PBS 0.1% tween 20 (Sigma) and proteins detected using SuperSignal West Pico Chemiluminescent substrate (Thermo Scientific).

Purified proteins were resolved by SDS-PAGE under reducing conditions using NuPAGE 4–12% Bis Tris precast gels (Invitrogen) and visualised by Coomassie Brilliant Blue R-250 (Sigma) staining.

### Protein purification and size exclusion chromatography

Proteins were purified from 300 ml of spent tissue culture supernatant by Nickel affinity chromatography on 1 ml His-Trap columns (GE Healthcare) following manufacturer's instructions using an ÄKTA Xpress (GE Healthcare). Purified proteins were further analysed by size exclusion chromatography on a Superdex 2000 Tricorn 10/600 column in HBS-EP buffer (GE Healthcare). Molecular masses were calculated based on the elution volumes compared to known protein standards (GE Healthcare).

## Results and Discussion

A short conserved domain was found near the N-terminus of our query protein (UniProtKB accession B0PET9.1), this domain is found in 1,403 sequence regions on 1,381 different proteins from 882 distinct species in UniProt release 2011_06 [6]. This is a large number of proteins for a completely unidentified domain, and so we were prompted to carry out a more in depth analysis. This domain was deposited in the Pfam database under the accession number PF09851. It was found to be homologous to the C-terminal 30 amino acids of a Pfam family previously called DUF2078 (Domain of Unknown Function 2078). The C-terminal 30 amino acids of DUF2078 were merged with this domain.

The SHOCT domain was so named because it is a short domain present at the C-terminus of many proteins (**SHO**rt **C**-**T**erminal domain). The entire domain is found within 50 amino acids of the C-terminus of the sequence in 89.2% of proteins in which it is found (in Pfam release 26.0).

**Figure 1:**
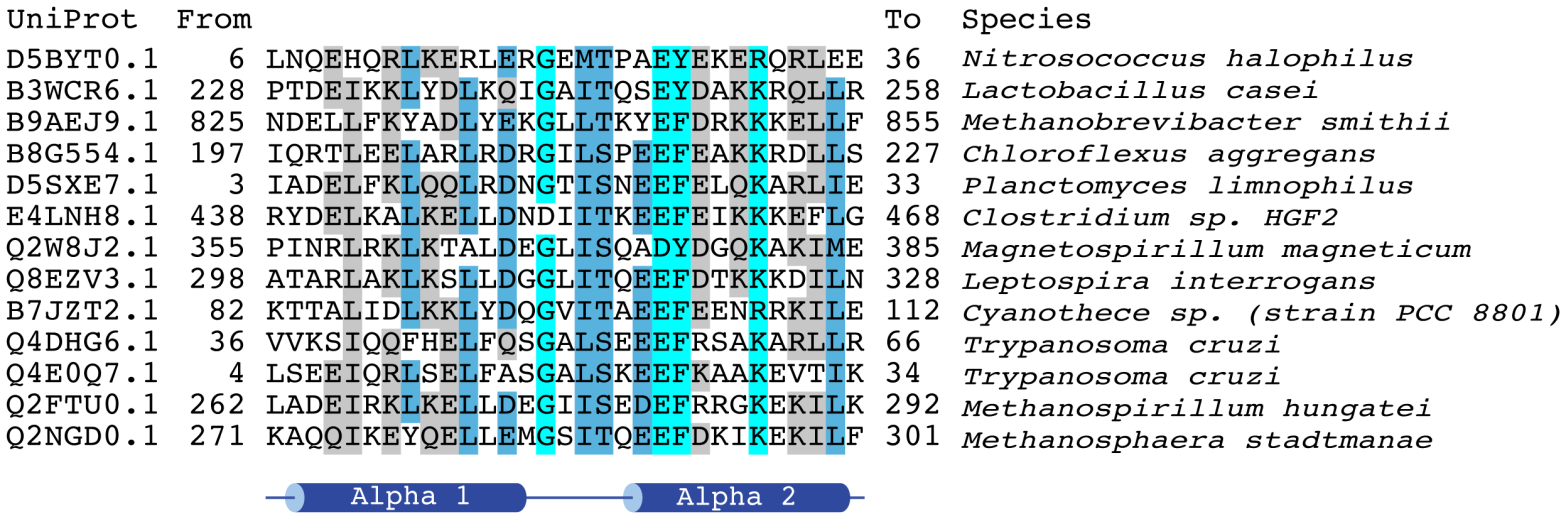
Multiple sequence alignment of SHOCT domains. Colouring by conservation has been produced using the belvu alignment viewer, conservation was calculated using the BLOSUM62 matrix. Secondary structure prediction was made using the Jpred3 web server [11].

**Figure 2:**
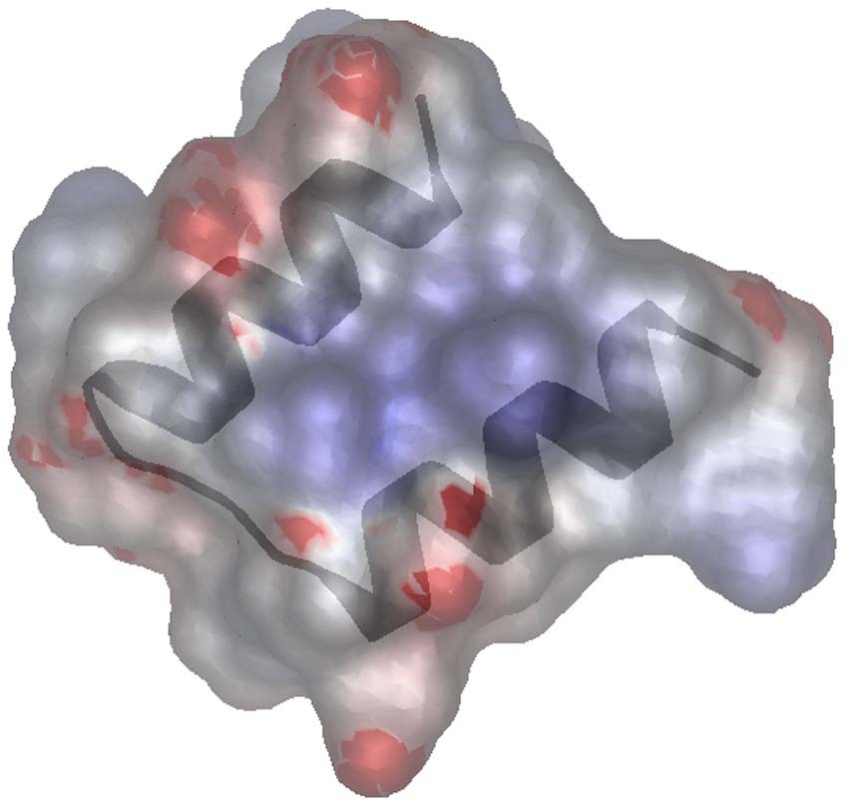
Three-dimensional structure prediction of the SHOCT domain of UniProtKB B0PET9.1 (residues 20–50) generated with QUARK using the default parameters[**12**] and viewed using MarkUs [**44**]. Surface electrostatic potential for the model is calculated using the program GRASP2 [45] accessed through MarkUs. The positively charged areas of the protein surface are shown in blue, and negatively charged areas in red, the two alpha helices are overlaid in grey.

The domain is around 30 amino acids in length and contains a strongly conserved glycine, a strongly conserved glutamic acid, two additional reasonably well-conserved charged amino acids, and five reasonably well conserved hydrophobic amino acids (figure 1). The secondary structure was predicted to contain two alpha helices using the Jpred3 server (http://www.compbio.dundee.ac.uk/www-jpred/) [11] using the Pfam SEED alignment (751 sequences). Using QUARK [12], it is predicted to fold into two amphipathic alpha helices (figure 2). No insertions or deletions are present in either of the two predicted alpha helices or the loop that joins the two alpha helices. This suggests that there is a strong selective constraint against insertion and deletion of residues within this domain.

We have termed this a domain, but we cannot rule out the possibility that SHOCT is in fact a motif. There are few structural domains that are as short as SHOCT, and those that do exist tend to be stabilised by disulphide bonds and/or interactions with metal ions as found in zinc finger domains. The lack of conserved cysteine and histidine residues in SHOCT indicates this this is not the case here. There are exceptions to this, one such short domain is the WW domain, which binds to proline-rich proteins[13]. The structure of the WW domain is stabilised by two highly conserved tryptophan residues.

**Figure 3:**
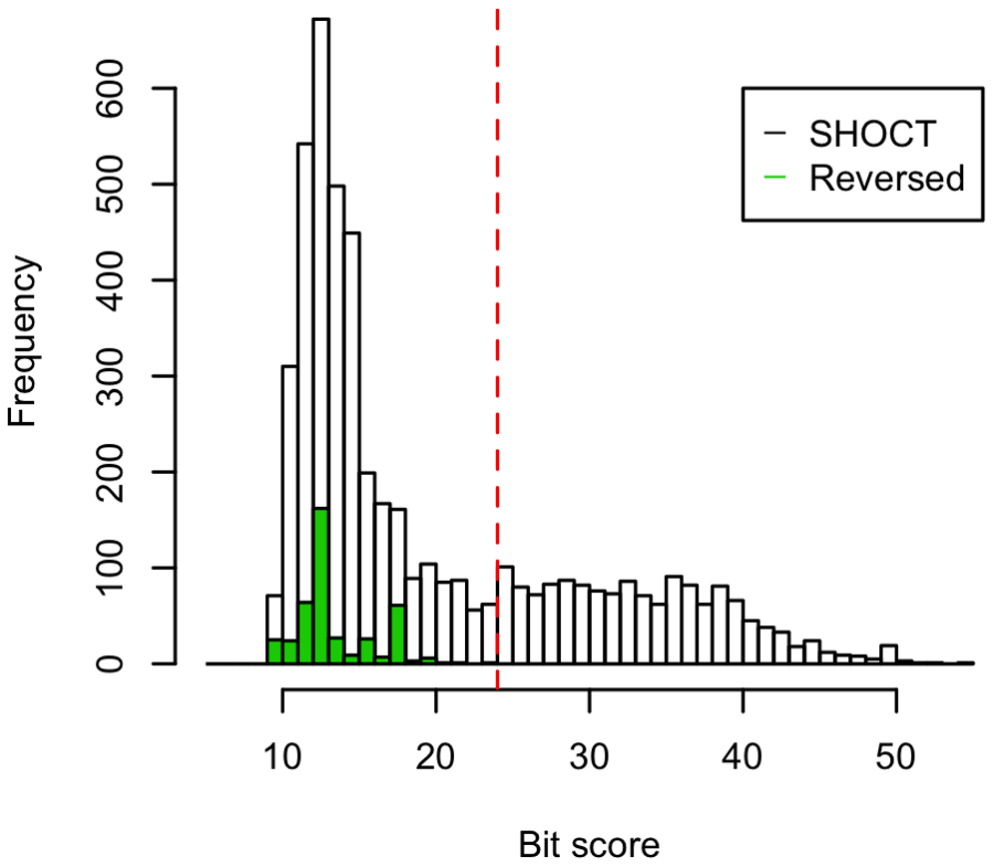
Histogram showing the bit scores distribution of the SHOCT domain HMM compared to a reversed version of the SHOCT domain HMM, searched against the UniProtKB database. The UniProtKB database was searched using an HMM constructed from the SHOCT seed alignment (unfilled bars) and an HMM from the reversed version of this alignment (green bars). The vertical line represents the sequence inclusion threshold.

As a control computational experiment to ensure that our HMM model was not identifying spurious matches, we searched the UniProtKB database with a reversed version of the SHOCT seed alignment using the same parameters. None of the hits to this reversed alignment had a bit score of greater than or equal to our selected sequence inclusion threshold of 24.0 bits (figure 3). There is an excess of hits below the inclusion threshold when the SHOCT seed alignment is used compared to the reversed version. This excess of matches may be caused by low-scoring related matches (false negatives).

### Species distribution

In Pfam release 26.0, the SHOCT domain is found in proteins from 882 distinct species. These are mainly bacteria (790 species) and archaea (53 species). It is widely distributed amongst both Gram-positive and Gram-negative bacteria, with 29.6% of the bacterial species being Firmicutes, 25.3% Proteobacteria, 19.1% Actinobacteria, 5.2% Bacteroidetes and 4.1% Cyanobacteria. Besides the bacteria and archaea, it is also found in proteins from 15 different bacteriophage species, 19 eukaryotic species and a small number of unclassified species from metagenomic studies. The eukaryotic species include trypanosomes, the amoeba *Naegleria gruberi*, and the slime mold *Dictyostelium discoideum*. The SHOCT domain is only found in one species of higher plant (*Ricinus communis*, the castor bean) and one insect species (*Culicoides sonorensis*). The *R. communis* protein SHOCT is found in, UniProtKB accession B9TKH1.1, only has similarity to bacterial proteins and not to other eukaryotic proteins. It is likely that this is not a true plant protein and that it arises from bacterial contamination. The insect protein is a fragment arising from a translation of an expressed sequence tag (EST), and has low similarity to any other proteins in UniProtKB.

The species distribution of the SHOCT domain is surprising in that it appears to be absent from proteins produced by well-characterised species, despite being present in closely related species. This may explain why it has remained unidentified until now. For example it is present in several species of *Bacillus* including one strain of *B. subtilis*, but is not found in *Bacillus subtilis* strain 168[14], it is present in a small number of *Pseudomonas* species, but absent from *Pseudomonas aeruginosa*. Of the 23 bacterial model organisms analysed by Hedges (2002), the SHOCT domain is only present in 6 (Table 1)[15]. Several of these species have numerous completely sequenced strains and, as in the case of *B. subtilis*, SHOCT is present in some of the sequenced strains and absent from others. There are a couple of possible explanations for this species distribution: the domain may have been acquired by horizontal transfer between different species, or it may have been initially present in the ancestors of many more species and subsequently lost from these species. In order to check that the apparent absence of the SHOCT domain from these organisms was not an artefact caused by proteins falling just below the inclusion threshold we selected 100 SHOCT domain sequences at random and performed iterative searches on these using Jackhmmer with default parameters. Several potential new SHOCT domains were discovered by this method, on average 3.9% of the sequences identified by each Jackhmmer search were new. However, these did not include any potential SHOCT domains from model organisms.

**Table 1:** Absence of the SHOCT domain from most model organisms.

Species	SHOCT domain?
*Escherichia coli*	No
*Salmonella typhimurium*	No
*Haemophilus influenzae*	No
*Vibrio cholera*	No
*Pseudomonas aeruginosa*	No
*Neisseria meningitidis*	No
*Rickettsia prowazekii*	No
*Helicobacter pylori*	No
*Synechocystis* spp.	Yes
*Deinococcus radiodurans*	No
*Streptomyces coelicolor*	Yes
*Mycobacterium tuberculosis*	Partial*
*Ureaplasma urealyticum*	No
*Mycoplasma pneumoniae*	No
*Streptococcus pneumoniae*	No
*Staphylococcus aureus*	Partial*
*Bacillus subtilis*	Partial*
*Thermotoga maritima*	Yes
*Aquifex aeolicus*	No
*Chlamydophila pneumoniae*	No
*Chlamydia trachomatis*	No
*Treponema pallidum*	No
*Borrelia burgdorferi*	No

* SHOCT is found in *Bacillus subtilis* strain BSn5, but not the more commonly studied strain 168, it is found in some strains of *Mycobacterium tuberculosis*, but not in the commonly studied strain H37Rv, it is found in some strains of *Staphylococcus aureus* but not the well characterised MRSA252, MSSA476, EMRSA-15, MSHR1132 and LGA251 strains.

### Domain architectures

Domains co-occurring on the same protein chain have been shown in some cases to be functionally related[16]. Therefore the study of the different domain architectures a domain participates in may provide important clues to its function. In order to elucidate the potential function of the SHOCT domain we have identified other protein domains present on proteins containing the SHOCT domain. The SHOCT domain is present in proteins containing 63 different domain architectures in Pfam 26.0 (Table S3).

### Oligomeric proteins containing a SHOCT domain

**Figure 4:**
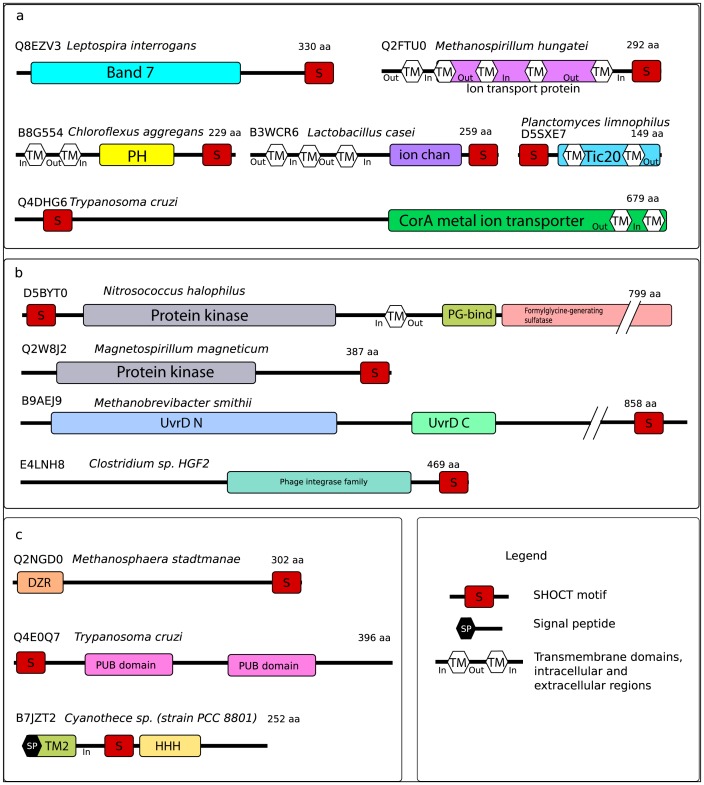
Domain architectures of selected proteins containing the SHOCT domain. Panel a shows proteins which are likely to be oligomeric, panel b shows enzymes and panel c shows binding proteins. Signal peptide and transmembrane domains are predicted using the Phobius web server [46].

The SHOCT domain is associated with many domains found in oligomeric proteins (figure 4a). It is most commonly associated with the band 7 domain (PF13421), this domain belongs to the SPFH superfamily (which is so-called because it includes **s**tomatins, **p**rohibitins, **f**lotillins and **H**flK/C proteins). The band 7 proteins are physiologically important proteins found in both prokaryotes and eukaryotes[17]–[19]. These proteins have been shown to oligomerise [19], in the case of stomatin the cytoplasmic C-terminus of the protein has been shown to be essential for oligomerisation [20]. Another domain structurally related to the band 7 domain is the major vault protein shoulder domain (PF11978). The major vault proteins form oligomers via hydrophobic interactions between alpha helical domains near to the C-terminus of the protein [21].

Pleckstrin homology (PH) domains are found in a large number of eukaryotic proteins involved in intracellular signalling and cytoskeletal regulation [22]. PH-like domains have also been identified in bacterial proteins that have been shown to form oligomeric rings [23]. In some Bacillales a short alpha-helical domain at the C-terminus of the PH-like domain mediates oligomer formation [23]. The SHOCT domain co-occurs with a bacterial PH domain (PF03703, previously known as DUF304) in nine proteins.

There are nine Trypanosome sequences in which SHOCT domain is present with a CorA domain (PF01544), in these sequences the SHOCT domain occurs at the N-terminus. CorA proteins are divalent metal ion transporters. In bacteria, CorA forms a funnel-shaped pentameric structure with two transmembrane helices towards the C-terminus of the protein [24].

The SHOCT domain is present at the C-terminus of three proteins with an ion channel domain (PF07885). The structure of a protein containing an ion channel domain from *Rhizobium loti* reveals that it is a homotetramer comprised of two dimers. Each subunit contains transmembrane helices in the N-terminal half of the protein and a cyclic nucleotide-binding domain (PF00027) at the C-terminus. The cyclic nucleotide-binding domain is often associated with ion channels, in the *R. loti* protein this domain appears likely to mediate dimerisation [25]. *Rattus norvegicus* small-conductance Ca2+-activated K+ channel (SK2) is an ion channel which includes a central ion channel domain, this protein contains a calmodulin-binding domain (CaMBD, PF02888) at the C-terminus. CaMBD consists of two long alpha-helices, which form dimers [26].

The SHOCT domain is also present at the C-terminus of one protein in an ion transport family (PF00520). This family is in the same clan as the ion channel domain (PF07885). The human voltage-gated hydrogen channel 1 (HV1) is a member of the ion transport family. This protein dimerises through interactions between coiled coils at its C-terminus [27]. In many cases proteins belonging to this family contain a tetramerisation domain (PF02214) at their N-terminus, which mediates tetramer formation and channel gating [28].

In addition to ion transport channels, SHOCT domains are also associated with protein transport channels. Tic20 (PF09685) is a channel protein that forms a part of the Tic complex, which is responsible for protein precursor import into chloroplasts [29]. The SHOCT domain is found at the N-terminus of 12 Tic20-containing proteins in species of Bacteroidetes, Planctomycetes and Proteobacteria.

**Figure 5:**
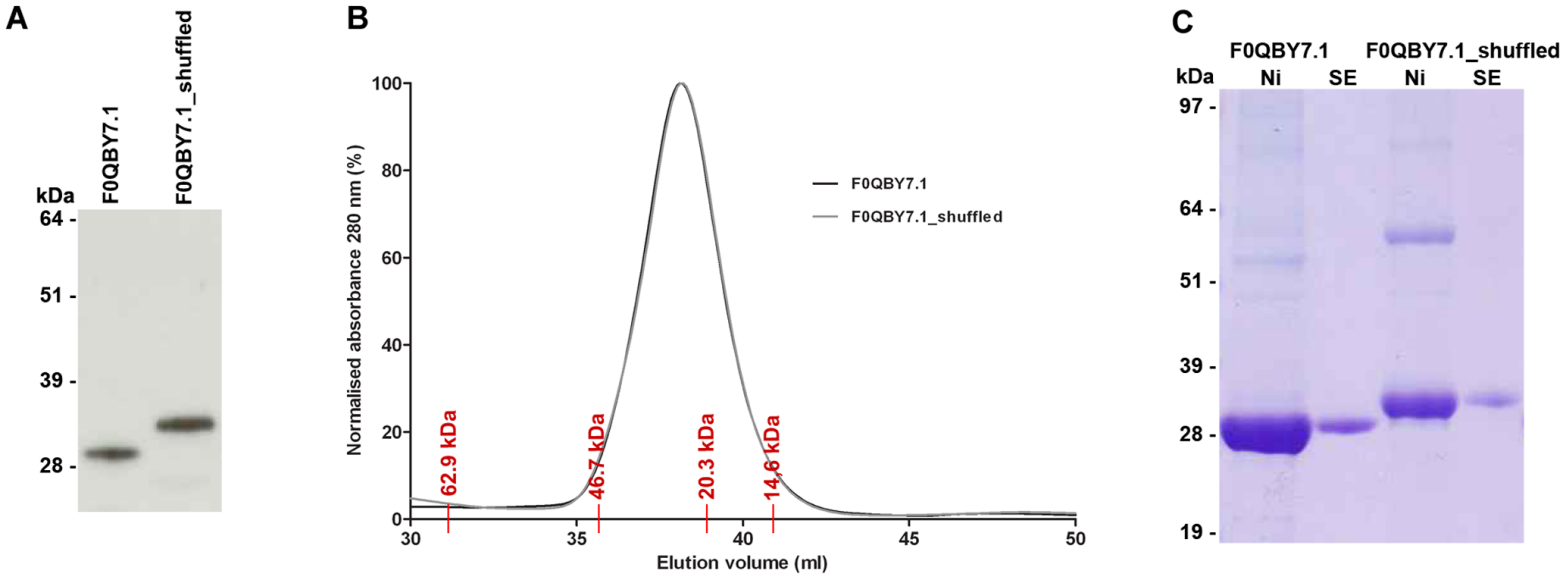
The SHOCT peptide does not multimerise the rat Cd4d3+4 protein. (**A**) Tissue culture supernatants containing biotinylated Cd4d3+4-F0QBY7.1 and Cd4d3+4-F0QBY7.1_shuffled were resolved by SDS-PAGE under reducing conditions, blotted and detected using streptavidin-HRP. (**B**) Purified Cd4d3+4-F0QBY7.1 and Cd4d3+4-F0QBY7.1_shuffled were resolved on a Superdex 2000 Tricorn 10/600 column. The elution volumes of protein standards are marked in red. The expected monomer size for Cd4d3+4-peptide is 33 kDa. (**C**) Purified Cd4d3+4-F0QBY7.1 and Cd4d3+4-F0QBY7.1_shuffled were resolved by SDS-PAGE under non-reducing conditions before (Ni) and after size exclusion chromatography (SE) and detected Coomassie Brilliant Blue R-250 staining.

The above examples include proteins where short alpha helical domains or motifs at the C-termini are responsible for oligomer formation, including stomatin, major vault protein and the CorA ion transport family[20], [21], [24]. In addition, the SHOCT domain overlaps with a predicted coiled coil in 91 proteins. Coiled coils are alpha-helical motifs that mediate oligomerisation in functionally diverse proteins [30]–[32]. We therefore hypothesised that SHOCT may function as an oligomerisation domain, the amphipathic nature of the helices suggests that it may bind to itself. In order to test this hypothesis we expressed a representative SHOCT domain (UniProtKB F0QBY7.1 amino acids 316–346) fused to the C-terminus of domains 3 and 4 of the rat CD4 protein (Cd4d3+4), and as a control a randomly shuffled version of this SHOCT domain fused to Cd4d3+4 (Table S2). Size exclusion chromatography was used to calculate the masses of these proteins. The proteins migrated at the size expected for the monomeric Cd4d3+4-SHOCT fusion protein, demonstrating that the SHOCT domain does not multimerise this protein construct (figure 5).

### Enzymes containing a SHOCT domain

The SHOCT domain is found in several different enzymes (figure 4b). One protein contains an N-terminal SHOCT domain, followed by a protein kinase domain (PF00069), a peptidoglycan binding domain (PF01471) and a C-terminal formylglycine generating sulfatase domain (PF03781). The *Chlamydia trachomatis* protein kinase pkn1 contains both an N-terminal protein kinase and a C-terminal formylglycine generating sulfatase domain. This enzyme has been shown to interact with, and phosphorylate, the membrane protein IncG and may play a role in host-pathogen interactions [33].

Several other kinases contain a SHOCT domain. A predicted serine/threonine kinase from the proteobacterium *Magnetospirillum magneticum* contains a protein kinase domain (PF00069) and a C-terminal SHOCT domain. The protein kinase domain is found in association with numerous different domains. Amongst the most commonly associated domains are the PASTA domain (PF03793), which binds to the beta-lactam rings of antibiotics [34], and the calcium-binding EF hand domain (PF13499). The SHOCT domain is also found in two oomycete kinases from the alpha-kinase family (PF02816), this eukaryotic family of protein kinases appears to be unrelated to the other protein kinase families [35]. The SHOCT domain is also found in several other enzymes including UvrD/Rep family helicases (PF00580), phage integrase (PF00589), and the putative peptidase family M60-like (PF13402).

The SHOCT domain is often found associated with the phospholipase D-nuclease N-terminal domain (PF13396), which is frequently found at the C-terminus of cardiolipin synthetase enzymes. The probable active site of these enzymes is not in the phospholipase D-nuclease N-terminal domain but is likely to be in a duplicated PLD-like domain (PF00614) [36]. The PLD-like domain is not found in association with the SHOCT domain, and the 80 proteins that contain the phospholipase D-nuclease N-terminal domain and the SHOCT domain are no larger than 173 amino acids in length implying that these proteins are not likely to include an additional catalytic domain.

### Binding proteins containing a SHOCT domain

The SHOCT domain is found in association with several different domains that are involved in binding to proteins and binding to DNA (figure 4c).

It is found in a number of putative DNA-binding proteins. In eight cases it occurs at the C-terminus of proteins containing the double zinc ribbon domain (PF12773). This domain is a member of the zinc-beta ribbon superfamily [37]. Zinc ribbons, which are present in a wide range of proteins, are usually stabilised by zinc ions and can bind nucleic acids and proteins [38]–[40].

Two proteins containing the SHOCT domain have a TM2 domain (PF05154) at the N-terminus, a SHOCT domain in the centre of the protein and a helix-hairpin-helix motif (PF12836) at the C-terminus. The TM2 domain consists of a short pair of transmembrane alpha-helices connected by a short linker, the biological function of this domain is unknown. The helix-hairpin-helix motif is a short DNA-binding motif [35]. Like the SHOCT domain, the helix-hairpin-helix is a short domain consisting of two alpha helices separated by just a few amino acids. A closely related helix-hairpin-helix motif (PF00633) is found in the DNA integrity scanning protein, DisA, from *Thermotoga maritima*. DisA functions as a diadenylate cyclase. It consists of three domains: a globular N-terminal nucleotide binding domain (PF02457), which binds a cyclic diadenosine phosphate, a central alpha-helical domain (the helical spine) (PF10635) and a C-terminal helix-hairpin-helix motif (PF00633). DisA forms octamers, the crystal structure of *T. maritima* DisA reveals that the formation of octamers is largely mediated by interactions between two of the alpha helices in the helical spine [41].

In addition to DNA-binding domains, the SHOCT domain is also found in several proteins that contain protein-binding domains. Amongst these are three proteins that contain an N-terminal SHOCT domain followed by a duplicated PUB domain (PF09409), and one protein containing a PUB domain and a C-terminal SHOCT domain. PUB domains, which are also known as PUG domains, are found in peptide-N-glycanases where they bind to the AAA ATPase p97 [42].

### SHOCT domains in Naegleria gruberi

Although most SHOCT domain-containing proteins only include one copy of SHOCT there are several proteins that contain multiple copies. The SHOCT domain is found in 14 proteins from the amoeba *Naegleria gruberi*, in the majority of these proteins one SHOCT domain is present, usually at the C-terminus. Two proteins from *Naegleria gruberi* contain three copies of the SHOCT domain, and one protein (UniProtKB accession D2VS68.1) contains eight repeats of the SHOCT domain. In these proteins containing multiple SHOCT domains the repeats are not identical.

## Conclusions

We have identified a new protein domain that is present in proteins with various functions from a wide range of predominantly bacterial species. We have named this the SHOCT domain. Its widespread distribution suggests that it may have an important function. It is surprising that it has not been discovered previously, but this may be because it is missing from well-studied species. One possible hypothesis for the function of this domain is as an oligomerisation domain, we tested this hypothesis experimentally and found that it did not appear to function as an oligomerisation domains.

Another possible hypothesis for the function of the SHOCT domain is that it may bind to something other than itself. It may bind to other protein domains/motifs or to nucleic acid. It's secondary structure is reminiscent of the helix-hairpin-helix motif, with both consisting of a pair of alpha helices separated by a small number of amino acids. It is therefore possible that SHOCT is a distant evolutionary relative of other helix-hairpin-helix proteins and may therefore bind to DNA.

Alternatively, SHOCT may function as a localisation domain. Its predominantly C-terminal location is similar to that of the Gram-positive cell wall sorting LPXTG motif, which is required for the anchoring of surface proteins to the bacterial cell wall [43]. We hope that this work encourages further research into this widespread yet enigmatic new protein domain.

## Supporting Information

Table S1 **Sequences of expressed peptides.**
(DOCX)

Table S2 **Primer sequences.** The underlined nucleotides represent the *AscI* and *NotI* restriction sites.
(DOCX)

Table S3 **Domain architectures of SHOCT domain-containing proteins.**
(DOCX)
