# Supplementary material for: The SHOCT Domain: A Widespread Domain Under-Represented in Model Organisms
Source: PLoS One. 2013 Feb 25;8(2):e57848. doi: 10.1371/journal.pone.0057848 (PMC3581485; doi:10.1371/journal.pone.0057848)
Supplement: Table S1 — Sequences of expressed peptides. (DOCX) [file pone.0057848.s001.docx]

| **Peptide** | **Sequence** |
| --- | --- |
| F0QBY7.1 | VMATLEKLGDLKAKGILTQEEFDAKKAELLK |
| F0QBY7.1_shuffled | DALKATLTEKAAEGKEDKVIELLFKQMLKGL |
