# Supplementary material for: The SHOCT Domain: A Widespread Domain Under-Represented in Model Organisms
Source: PLoS One. 2013 Feb 25;8(2):e57848. doi: 10.1371/journal.pone.0057848 (PMC3581485; doi:10.1371/journal.pone.0057848)
Supplement: Table S2 — Primer sequences. The underlined nucleotides represent the AscI and NotI restriction sites. (DOCX) [file pone.0057848.s002.docx]

| **Primer** | **Sequence** |
| --- | --- |
| F0QBY7.1 Forward | GGCCGCCGTGATGGCCACCCTGGAGAAGCTGGGCGACCTGAAGGCCAAGGGCATCCTGACCCAGGAGGAGTTCGACGCCAAGAAGGCCGAGCTGCTGAAGGG |
| F0QBY7.1 Reverse | CGCGCCCTTCAGCAGCTCGGCCTTCTTGGCGTCGAACTCCTCCTGGGTCAGGATGCCCTTGGCCTTCAGGTCGCCCAGCTTCTCCAGGGTGGCCATCACGGC |
| F0QBY7.1_shuffled Forward | GGCCGCCGACGCCCTGAAGGCCACCCTGACCGAGAAGGCCGCCGAGGGCAAGGAGGACAAGGTGATCGAGCTGCTGTTCAAGCAGATGCTGAAGGGCCTGGG |
| F0QBY7.1_shuffled Reverse | CGCGCCCAGGCCCTTCAGCATCTGCTTGAACAGCAGCTCGATCACCTTGTCCTCCTTGCCCTCGGCGGCCTTCTCGGTCAGGGTGGCCTTCAGGGCGTCGGC |
