# Supplementary material for: The SHOCT Domain: A Widespread Domain Under-Represented in Model Organisms
Source: PLoS One. 2013 Feb 25;8(2):e57848. doi: 10.1371/journal.pone.0057848 (PMC3581485; doi:10.1371/journal.pone.0057848)
Supplement: Table S3 — Domain architectures of SHOCT domain-containing proteins. (DOCX) [file pone.0057848.s003.docx]

| Domain architecture | Number of sequences |
| --- | --- |
| Band_7_1~SHOCT | 121 |
| PLDc_N~SHOCT | 80 |
| TM2~SHOCT | 33 |
| SHOCT~Tic20 | 12 |
| DUF304~SHOCT | 9 |
| SHOCT~CorA | 9 |
| DZR~SHOCT | 8 |
| Imm_superinfect~SHOCT | 7 |
| PPE~PE_PPE_C~SHOCT | 5 |
| SHOCT~SHOCT | 5 |
| DUF697~SHOCT | 5 |
| SHOCT~PUB~PUB | 3 |
| DUF4126~SHOCT | 3 |
| Ank_2~Ank_2~Ank_2~Ank_2~Ank_2~SHOCT | 3 |
| Ion_trans_2~SHOCT | 3 |
| PEGA~SHOCT | 3 |
| CH~SHOCT | 3 |
| SHOCT~Alpha_kinase | 2 |
| DUF2846~SHOCT | 2 |
| TM2~SHOCT~HHH_3 | 2 |
| Tubulin_2~SHOCT | 2 |
| SHOCT~SHOCT~SHOCT | 2 |
| UvrD-helicase~UvrD_C~SHOCT | 2 |
| DUF805~SHOCT | 2 |
| SHOCT~DUF4190 | 1 |
| DUF4234~SHOCT | 1 |
| F420_oxidored~SHOCT | 1 |
| Ank_2~Ank_2~Ank_2~Ank_2~Ank~Ank_2~SHOCT | 1 |
| HAMP~SHOCT | 1 |
| Ion_trans~SHOCT | 1 |
| SHOCT~Apc3~TPR_1~TPR_11~TPR_2~TPR_1~TPR_11~TPR_16~TPR_11~TPR_11~TPR_11 | 1 |
| SHOCT~TPR_2 | 1 |
| DUF350~Band_7_1~SHOCT | 1 |
| TPR_16~SHOCT~SPOR | 1 |
| PhageMin_Tail~SHOCT | 1 |
| SHOCT~PspC | 1 |
| DUF3592~SHOCT | 1 |
| Copper-bind~SHOCT | 1 |
| YfiO~SHOCT | 1 |
| SHOCT~PUB | 1 |
| M60-like~SHOCT | 1 |
| Pkinase~SHOCT | 1 |
| SHOCT~SHOCT~TM2 | 1 |
| HEAT_2~SHOCT | 1 |
| Cupin_2~SHOCT~SHOCT~SHOCT~SHOCT | 1 |
| Band_7~SHOCT | 1 |
| SHOCT~zf-ribbon_3 | 1 |
| zinc_ribbon_2~SHOCT | 1 |
| Mrr_N~SHOCT | 1 |
| SHOCT~Pkinase~PG_binding_1~FGE-sulfatase | 1 |
| CH~SHOCT~SHOCT~SHOCT | 1 |
| Ax_dynein_light~SHOCT | 1 |
| SHOCT~VHP | 1 |
| Actin~SHOCT | 1 |
| SHOCT~SHOCT~SHOCT~SHOCT~SHOCT~SHOCT~SHOCT~SHOCT | 1 |
| SHOCT~DUF4355 | 1 |
| Ank_2~Ank_2~Ank_2~Ank_2~SHOCT | 1 |
| Response_reg~SHOCT | 1 |
| DUF4064~SHOCT | 1 |
| Phage_integrase~SHOCT | 1 |
| Phage_HK97_TLTM~SHOCT | 1 |
| SHOCT~SAP~DnaJ | 1 |
